# FGF21 Protects Against Hypoxia Injury Through Inducing HSP72 in Cerebral Microvascular Endothelial Cells

**DOI:** 10.3389/fphar.2019.00101

**Published:** 2019-02-20

**Authors:** Hao-Wei Wang, Xin Jiang, Yu Zhang, Jian Wang, Jian Xie, Yong-Qiang Wang, Yong-Hua Li

**Affiliations:** ^1^ Department of Anesthesiology, Changzheng Hospital, Second Military Medical University, Shanghai, China; ^2^ Department of Anesthesiology, Shuguang Hospital Affiliated to Shanghai University of Traditional Chinese Medicine, Shanghai, China

**Keywords:** FGF21, hypoxia, heat shock protein family A member 1A, cerebral microvascular endothelial cell, cyclooxygenase-2, oxidative, NF-κB, matrix metalloprotein

## Abstract

**Background:** Fibroblast growth factor 21 (FGF21), a member of a family of atypical FGFs, functions as cytokine to control endocrinology and metabolism. Recently, the roles of FGF21 in cardio-cerebral-vascular diseases have been gradually uncovered. In the present study, we investigated the effect of FGF21 on bEnd.3 cerebral microvascular endothelial cells (CMECs) upon hypoxia stress.

**Methods and Results:** CMECs were cultured in the condition of 1% O_2_ for 8 h to induce hypoxia stimuli. For FGF21 treatment, recombinant FGF21 (50 nM) was added into the culture medium. Various biomedical assays were used to evaluate the hypoxia-induced injury in CMECs. Under normoxia condition, FGF21 had no obvious effect on cell viability and did not cause any cytotoxicity on CMECs. Under hypoxia condition, FGF21 significantly attenuated the hypoxia-induced injury, evidenced by the influences of FGF21 on CMEC viability and LDH release. TUNEL staining assay and immunoblotting of caspase-3 showed that FGF21 reduced hypoxia-induced apoptosis in CMECs. Mechanistically, FGF21 treatment compromised the hypoxia-induced changes of reactive oxygen species, malondialdehyde, total antioxidant activity, and total superoxide dismutase levels. FGF21 administration decreased hypoxia-induced matrix metalloprotein 3 and matrix metalloprotein 2/9 activity in CMECs. Activities of cyclooxygenase-2 and NF-κB-p65, two pro-inflammatory factors, were also upregulated by hypoxia but suppressed by FGF21. At last, we found that FGF21 increased heat shock protein family A member 1A (HSP72) mRNA and protein expression. Blockade of HSP72 by a pharmacological inhibitor VER155008 or specific siRNA-mediated knockdown abrogated the protection of FGF21 against hypoxia in CMECs.

**Conclusion:** These data demonstrate that FGF21 protects against hypoxia stress-induced injury in CMECs by inducing HSP72 expression, suggesting a therapeutic value of FGF21 in hypoxia-related brain diseases such as ischemic stroke and acute mountain sickness.

## Introduction

The well-known blood-brain barrier (BBB) is a crucial barrier, which impedes physical diffusion of most proteins or chemical compounds from blood to brain. The main function of BBB is to maintain homeostasis *via* selective controlling fluid and biomolecule exchange processes. Cerebral microvascular endothelial cells (CMECs) are the major components of BBB (Huber et al., 2001). CMECs have tight junctions (TJs), which are critical for maintaining the brain homeostasis and low permeability. Disruption of BBB integrity can be triggered by hypoxic condition that occurs in ischemic stroke, decreased perfusion pathologies, and high-altitude exposure, which always lead to brain edema (Mark and Davis, 2002). Although there are numerous basic science studies and clinical investigations, the effect of hypoxia on cerebral microvasculature and the corresponding cellular mechanisms involved in the BBB disruption remain not fully elucidated.

Fibroblast growth factors (FGFs) are a group of naturally occurring heparin-binding proteins that are potent mitogens and chemoattractants for various cells. FGF21 is a member of the endocrine branch FGF subfamily and expressed mainly in several metabolically active tissue organs, such as the liver, thyroid, adipose tissue, skeletal muscle, and pancreas (Staiger et al., 2017). Unlike other prototypical members of FGFs, the mitogenic activity is absent in FGF21 (Fisher and Maratos-Flier, 2016). Alternatively, FGF21 exhibits pronounced regulatory functions on endocrinology and metabolism (Fisher and Maratos-Flier, 2016). FGF21 regulates PPARγ activity and is required for the anti-diabetic actions of thiazolidinediones (Dutchak et al., 2012). Administration of FGF21-mimetic antibody treats diabetes and obesity FGF21 by lowering blood glucose levels and enhancing insulin sensitivity in diabetic (Foltz et al., 2012; Holland et al., 2013). FGF21 reduces plasma triglyceride concentrations by accelerating lipoprotein catabolism in white and brown adipose tissues (Schlein et al., 2016).

In recent years, FGF21 has been found to play important roles in cardio-cerebral-vascular diseases. FGF21 prevented atherosclerosis by suppression of hepatic sterol regulatory element-binding protein-2 and induction of adiponectin (Lin et al., 2015) and treated angiotensin II-induced hypertension/vascular dysfunction (Pan et al., 2018). Importantly, it is noted that lyophilized FGF21 protected cerebral ischemia in middle cerebral artery occlusion (MCAO) rats and neuronal cells *via* decreasing endoplasmic reticulum stress (Yang et al., 2018). However, whether FGF21 is involved in BBB disruption hypoxia-induced injury has not been fully elucidated. Our hypothesis is whether FGF21 attenuates hypoxia-induced injury in cultured cerebral microvascular endothelial cells. In the present study, we test this hypothesis in bEnd.3 mouse CMEC cell line *in vitro*. Our findings indicate that FGF21 protects against hypoxia-induced injury in CMECs through inducing heat shock protein family A member 1A (HSP72), a stress-inducible ATPase molecular chaperone.

## Materials and Methods

### Reagents

Recombinant FGF21 was purchased from PeproTech Inc. (Rocky Hill, NJ, USA). Antibody against caspase-3 was obtained from Cell Signaling Biotechnology (Danvers, MA, USA). Antibodies against HSP72 and β-actin were purchased from Santa-Cruz Biotechnology (Santa-Cruz, CA, USA). Terminal deoxynucleotidyl transferase dUTP nick end labeling (TUNEL) assay kit was purchased from Cell Biolabs Inc. (San Diego, CA, USA). Cell viability assay (Cell Counting Kit-8) was from Dojindo Molecular Technologies, Inc. (Kumamoto, Japan). LDH cytotoxic kit was purchased from Promega (Madison, WI, USA). Assays for detecting reactive oxygen species (ROS), total antioxidant activity (T-AOC), malondialdehyde (MDA), and total-SOD (T-SOD) activity were purchased from Beyotime (Haimen, Jiangsu, China). Protease/phosphatase inhibitors were purchased from Pierce (Rockford, IL, USA). Cyclooxygenase-2 (COX-2) and MMP-3 activities assays were purchased from Biovision (Mountain View, CA, USA). Zinc protoporphyrin-9 (VER155008) and MMP-9 activity assay were purchased from Sigma (St. Louis, MO, USA). NF-κB-p65 transcription factor assay kit was purchased from Cayman Chemical (Ann Arbor, MI, USA).

### Cell Culture and Treatment

Cerebral microvascular endothelial cell line (bEnd.3) was purchased from the American Type Culture Collection (Manassas, VA, USA). Cells were cultured in DMEM plus 1,640 medium (1:1, all from Gibco) supplemented with 10% fetal bovine serum (FBS, Gibco) in an incubator containing 95% O_2_ and 5% CO_2_ (Song et al., 2018). For FGF21 treatment in bEnd.3 cells, recombinant FGF21 was dissolved in distilled water and added into the medium to achieve the final concentration at 50 nM. This dose was chosen on the basis of previous studies (Planavila et al., 2015; Xue et al., 2015). For blockade of HSP72 in cells, HSP72 inhibitor VER155008 was used. The final concentration of VER155008 was 50 μM, and the final DMSO concentration in the cell culture medium was below 0.5% in all experiments conducted. Vehicles were included as controls.

### Induction of Hypoxia

Hypoxia condition was conducted according to a previously described protocol (Wang et al., 2011). Cells at ∼80% confluence were incubated with vehicle or FGF21 and placed in a hypoxia incubator (1% O_2_, 5% CO_2_, 94% N_2_; Thermo Forma) for 8 h. An automated regulator with built-in flow meter and oxygen sensor was used to ensure and maintain the proper composition of gas mixture within the incubator. After hypoxia treatment, the cells were removed from the chamber and immediately lysed for various biochemical analyses.

### Cell Viability Assay

The cells were plated in 96-well plates and maintained in culture medium and subjected with hypoxia stress. At five time points after transfection, the cell viability was determined with a commercial Cell Counting Kit-8 as described previously (Wang et al., 2009). About 10 μL of CCK-8 solution was added into the medium. Cells were maintained at 37°C for 1 h. The optical density at 450 nm was obtained using a microplate reader (TECAN, Sweden) (Qin et al., 2017). Experiments were performed in duplicate. The interassay variation is <8%.

### LDH Release Assay

LDH release was determined using Promega CytoTox-ONE™ kit (Promega). After the hypoxia treatment, the cell medium was removed swiftly and transferred to a black fluorescence plate and incubated for 10 min with CytoTox-ONE™ reagent followed by stop solution. The fluorescence was measured at 560/590 nm. The release of LDH was calculated using the standard curve.

### TUNEL Assay

The cells were seeded in Nunc™ glass bottom dishes (Thermo) and treated as mentioned above. After hypoxia treatment, the cells were fixed in 4% paraformaldehyde for 25 min. The cells were then permeabilized with prechilled 0.2% Triton X-100/PBS for 5 min on ice. After permeabilization, TUNEL assay was performed according to the manufacturer’s instructions as described previously (Lin et al., 2017; Li et al., 2018a). The nuclei were stained by DAPI for 10 min. The cells were observed under a fluorescent microscopy (IX71; Olympus, Japan). The TUNEL-positive (green) cells per visual field were calculated to reflect the apoptosis.

### Quantitative Real-Time PCR

Total RNA was extracted from bEnd.3 cells by using TRIzol reagent (Invitrogen, CA, USA) and converted to cDNA using MMLV reverse transcriptase (Takara, Tokyo, Japan). For real-time PCR, SYBR qPCR Real-Time kit (Takara, Tokyo, Japan) was used according to the manufacturer’s instructions and amplified with the real-time PCR detection system (Bio-Rad). Amplification conditions were set as 40-cycle program (95°C for 15 s, 60°C for 30 s, 72°C for 45 s) (Zhou et al., 2017b). The mRNA level of HSP72 gene was normalized to β-actin, and the results were analyzed using 2^−△△*C*T^ method as described previously (Wang et al., 2017). The PCR primers to detect HSP72 mRNA used in qRT-PCR were 5′-GTGCGTGGGCGTGTTCC-3′ and 5′-CGGTGTTCTGCGGGTTCA-3′, respectively. The PCR primers used to detect β-actin were 5′-CAGCCACCCGAGATTGAGCA-3′ and 5′-TAGTAGCGACGGGCGGTGTG-3′, respectively.

### Immunoblotting

Immunoblotting was performed as described previously (Li et al., 2018b). The cells treated with hypoxia were washed by ice-cold PBS for three times and then lysed by RIPA buffer (Beyotime) supplemented with protease inhibitor and protein phosphatase inhibitors. The protein samples were boiled for 10 min and separated in 10% SDS-PAGE at 100 V. The proteins were then transferred at 4°C to a polyvinyldifluoride (PVDF) membrane (Bio-Rad, NJ, USA) for 1 h at a constant voltage of 400 mA in a transfer buffer containing 10% methanol. The nonspecific protein binding was blocked with 5% weight (w)/v nonfat milk and 0.1% v/v Tween-20 in Tris-buffered saline (TBS) for 4 h (Busceti et al., 2017). The membranes were incubated with primary antibodies diluted in TBS containing 0.1% v/v Tween-20 (TBST) overnight at 4°C. After washing four times with TBST buffer, the membranes were incubated with IRDye-labeled secondary antibody and detected using Odyssey infrared-imaging system (Li-cor, Lincoln, NE, USA).

### Oxidative Stress Measurements

For ROS determination, the cells seeded on 96 well/plates were treated with hypoxia with or without FGF21 for 8 h and then incubated with the dichloro-dihydro-fluorescein diacetate probe (Invitrogen, Eugene, OR, USA) for 30 min. Then, the cells were washed by ice-cold PBS for three times, and the fluorescence intensity was measured by a multiple-functional micro-plate reader (TECAN) as described previously (Li et al., 2017). For T-AOC, MDA, and T-SOD measurement, cells were lysed by RAPI buffer, and the samples were conducted according to the manufacturer’s instruction, respectively.

### Activities of MMPs, COX-2, and NF-κB-p65

Fluorescence assays were used to determine activities of MMP-3, MMP-2/9, COX-2, and NF-κB-p65. The cells seeded on 96 well/plates were treated with hypoxia with or without FGF21 for 8 h and then lysed with distilled water and then conducted according to the manufacturer’s instruction. To evaluate MMP-3 activity, the fluorescence was measured at 325/395 nm. To evaluate MMP-2/9 activity, the fluorescence was measured at 320/405 nm. To evaluate COX-2 activity, the fluorescence was measured at 535/587 nm.

Colorimetric assays were used to determine the transcriptional activity of NF-κB-p65 in nuclear extract. The cells seeded on 96 well/plates were treated with hypoxia with or without FGF21 for 8 h and then carried out with nuclear extraction kit (Beyotime, Haimen, China). The process was conducted according to the manufacturer’s instruction. The colorimetric readout at 450 nm was obtained. The transcriptional activity of NF-κB-p65 was calculated according to the standard curve.

### siRNA-Mediated Knockdown

The CMECs at 60% confluence in a 24-well plate were transfected with siRNA targeting HSP72 (Santa-Cruz Biotechnology, Santa-Cruz, CA, USA) or siRNA-scramble (siRNA-control) with Lipofectamine LTX Reagent (Invitrogen) and Opti-MEM TM (Invitrogen). To determine the knockdown efficiency, real-time PCR and immunoblotting analyses were performed to confirm the HSP72 downregulation at 3 days after transfection (Li et al., 2018c).

### Statistical Analysis

All of the statistical calculations were performed using the GraphPad Prism 5 software program. The data were expressed as mean ± SEM. All data sets were normally distributed. Student’s *t* test was used to compare two conditions, and a one-way ANOVA with Tukey’s correction was used for multiple comparisons. Statistical significance was set at *p* < 0.05.

## Results

### FGF21 Promotes Cell Survival Upon Hypoxia Stress in CMECs

**Figure 1:**
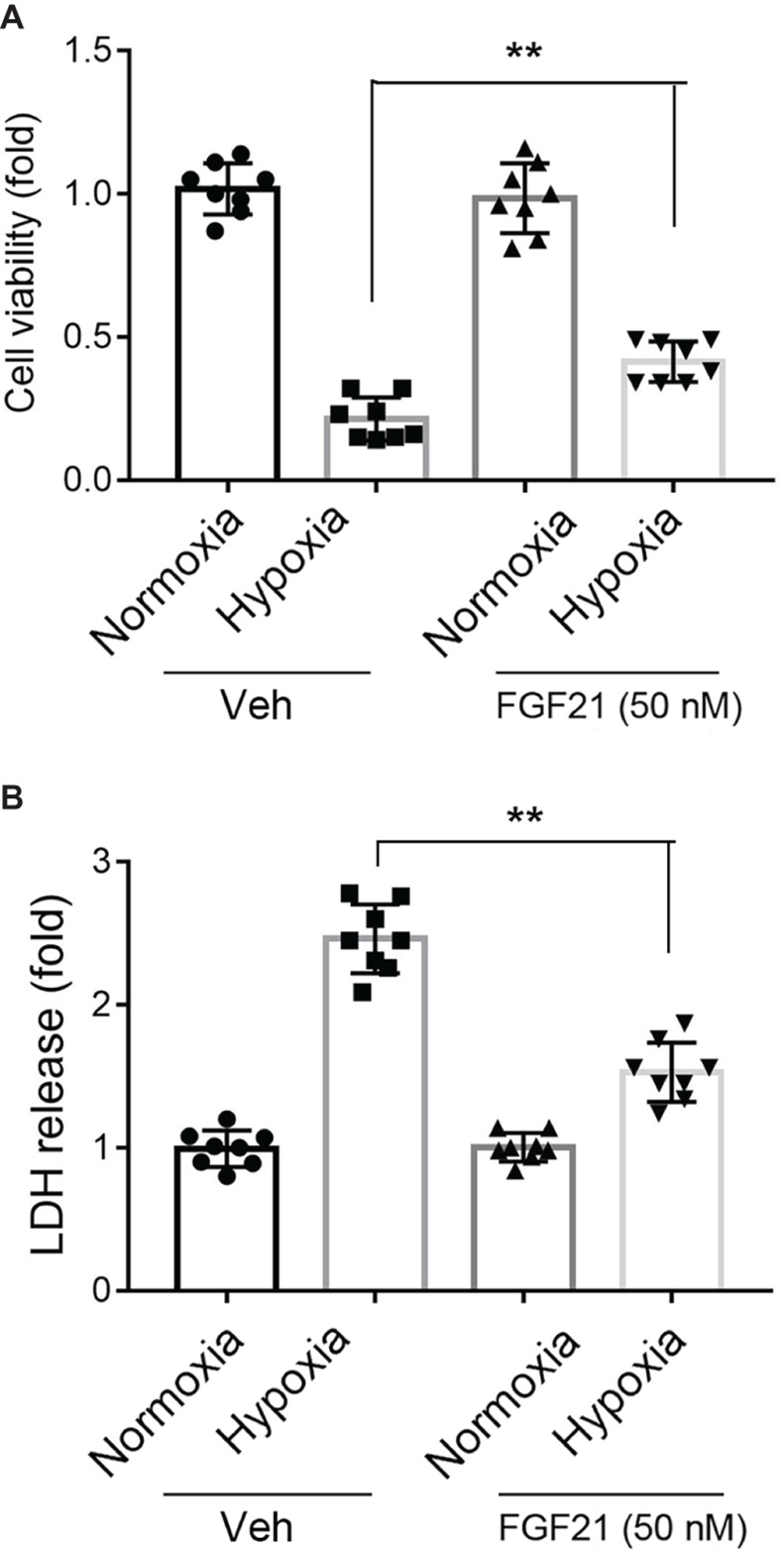
FGF21 promotes cell survival upon hypoxia stress in CMECs. **(A)** The cell viability of CMECs under the condition of normoxia and hypoxia (1% O_2_) with FGF21 treatment (50 nM) or vehicle for 8 h. **p < 0.01 vs. Veh. *N* = 8. **(B)** The LDH content in the culture medium of CMECs under the condition of normoxia and hypoxia (1% O_2_) with FGF21 treatment (50 nM) or vehicle for 8 h. **p < 0.01 vs. Veh. *N* = 8. The CMECs cultured in normoxia condition were also used as a control. Veh, vehicle. For *N* = 8, experiments were conducted in eight different wells of cultured cells.

Under normoxia condition, treatment of FGF21 (50 nM) had no obvious effect on CMECs viability and did not cause any cytotoxicity in CMECs (Figure 1A, black panels). As expected, the cell viability of CMECs was reduced to ~25% upon hypoxia for 8 h (Figure 1A). Strikingly, administration of FGF21 significantly attenuated the hypoxia-induced reduction of CMEC viability (*p* < 0.01; Figure 1A). The release of LDH, a marker of membrane integrity, was also determined. Recombinant FGF21 treatment significantly decreased hypoxia-induced LDH release in CMECs (*p* < 0.01; Figure 1B).

### FGF21 Decreases Hypoxia Stress-Induced Apoptosis in CMECs

**Figure 2:**
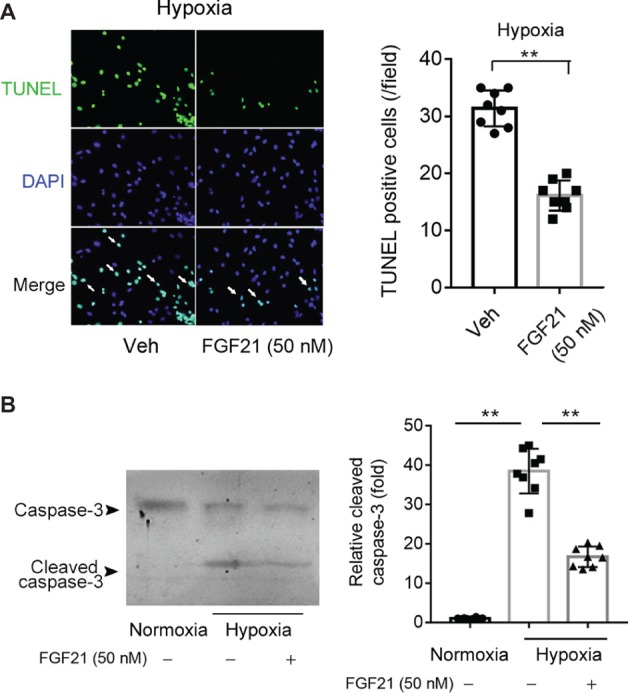
FGF21 decreases hypoxia stress-induced apoptosis in CMECs. **(A)** Representative images of fluorescent TUNEL staining in CMECs under the condition of normoxia and hypoxia (1% O_2_) with FGF21 treatment (50 nM) or vehicle for 8 h. **p < 0.01 vs. Veh. *N* = 8. **(B)** Representative images of immunoblotting assay of caspase-3 and cleaved caspase-3 in CMECs challenged by hypoxia (1% O_2_) with FGF21 treatment (50 nM) or vehicle for 8 h. **p < 0.01 *N* = 8. Veh, vehicle. For *N* = 8, experiments were conducted in eight different wells of cultured cells.

We next studied the effects of FGF21 on hypoxia-induced apoptosis in CMECs. TUNEL assay showed that hypoxia stimuli induced remarkable apoptosis in CMECs (Figure 2A). FGF21 partly but significantly prevented the apoptosis in CMECs upon hypoxia (*p* < 0.01; Figure 2A). We also measured cleaved caspase-3, a well-established molecular marker of apoptosis. As shown in Figure 2B, hypoxia significantly boosted the expression of cleaved caspase-3, which was substantially attenuated by FGF21 treatment. These results indicate that FGF21 decreases hypoxia-induced apoptosis in CMECs.

### FGF21 Suppresses Hypoxia-Induced Oxidative Stress in CMECs

**Figure 3:**
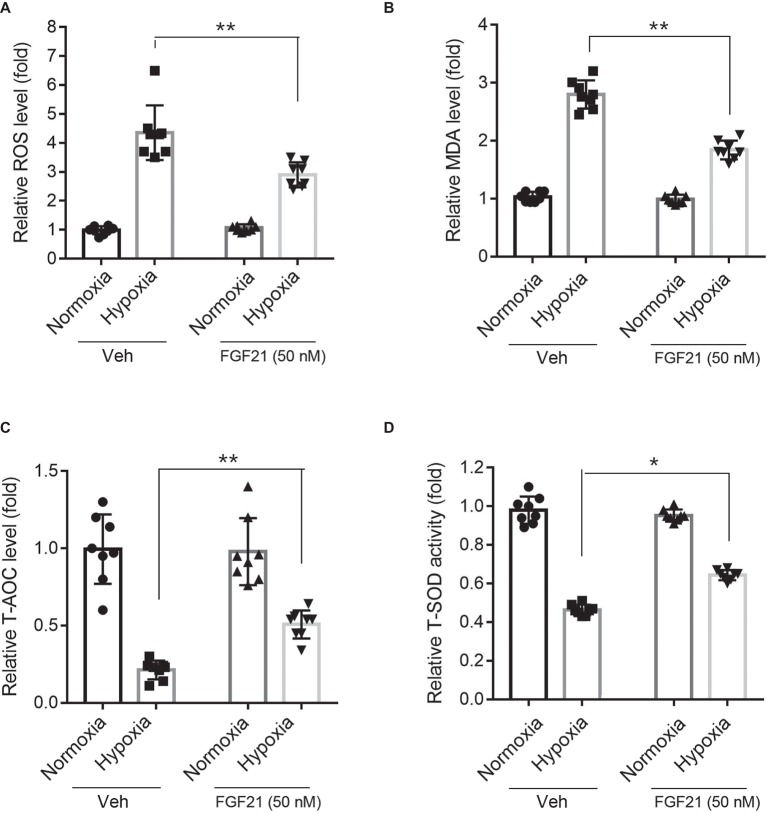
FGF21 suppresses hypoxia-induced oxidative stress in CMECs. **(A)** Quantitative analysis of intracellular ROS production in CMECs. **p < 0.01 vs. Veh. *N* = 8. **(B)** Quantitative analysis of intracellular MDA level in CMECs. **p < 0.01 vs. Veh. *N* = 8. **(C)** Quantitative analysis of intracellular total anti-oxidant activity (T-AOC) in CMECs. **p < 0.01 vs. Veh. *N* = 8. **(D)** Quantitative analysis of intracellular total SOD activity (T-SOD) in CMECs. *p < 0.05 vs. Veh. *N* = 8. Veh, vehicle. For *N* = 8, experiments were conducted in eight different wells of cultured cells.

Hypoxia treatment triggered ROS level in CMECs (Figure 3A). FGF21 significantly lowered the hypoxia-induced ROS level (*p* < 0.01; Figure 3A). Additionally, hypoxia challenge elevated the intracellular MDA level, a critical index of lipid peroxidation, which was partly lowered by FGF21 administration (*p* < 0.01; Figure 3B). We also measured two anti-oxidative factors. The T-AOC level in CMECs was significantly reduced by hypoxia but rescued partly by FGF21 treatment (*p* < 0.01; Figure 3C). Similarly, the level of T-SOD activity, a major scavenger of ROS, was markedly suppressed after hypoxia in CMECs, while FGF21 partly recovered the T-SOD activity (*p* < 0.05; Figure 3D). These results suggest that FGF21 suppresses hypoxia-induced oxidative stress in CMECs.

### FGF21 Decreases Hypoxia-Induced MMP Activities in CMECs

**Figure 4:**
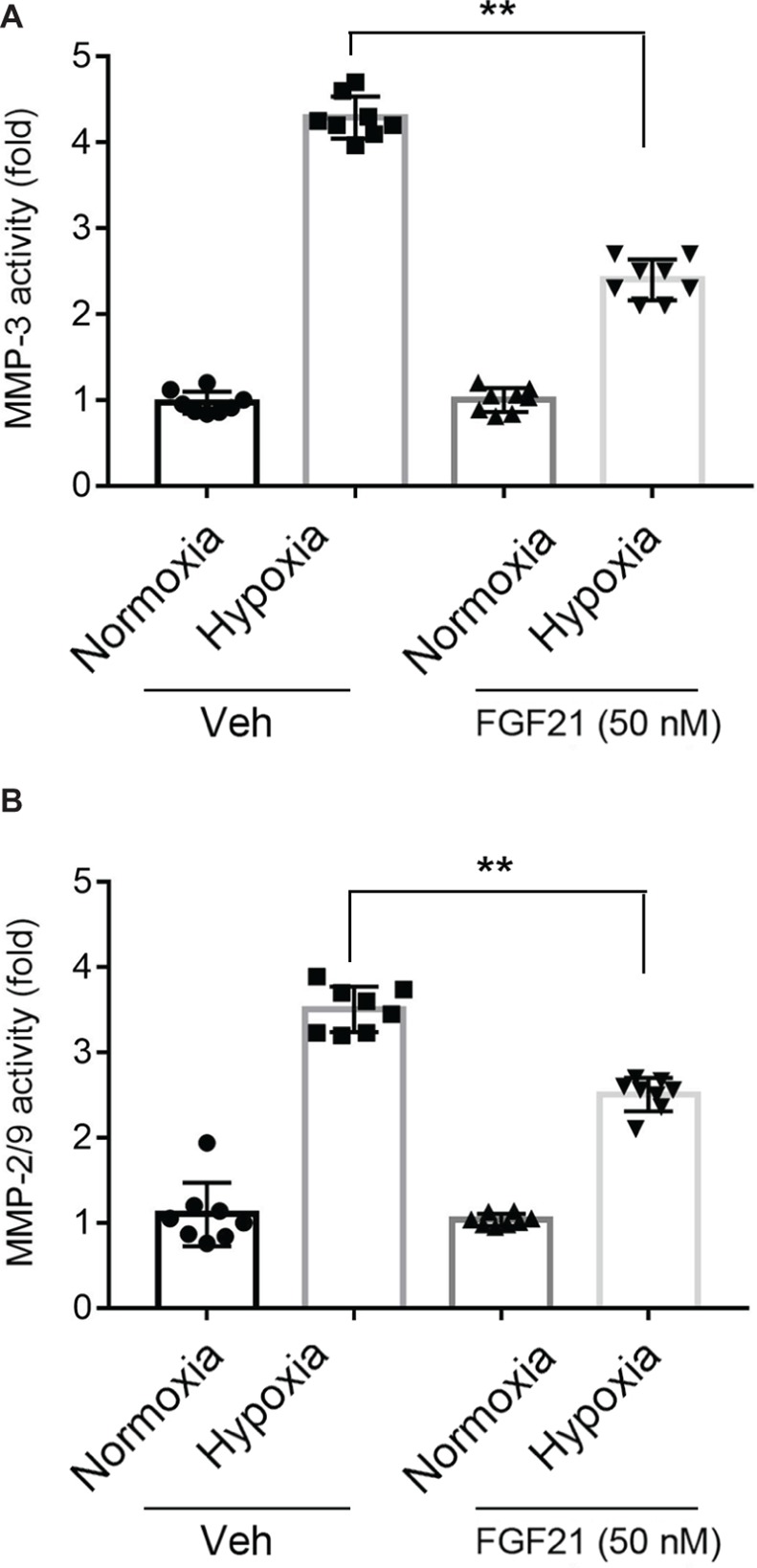
FGF21 decreases hypoxia-induced MMP activities in CMEC. **(A,B)** Quantitative analyses on the activities of MMP-3 **(A)** and MMP-2/9 **(B)** in CMECs under the condition of normoxia and hypoxia (1% O_2_) with FGF21 treatment (50 nM) or vehicle for 8 h. *p < 0.05, **p < 0.01 vs. Veh. *N* = 8. Veh, vehicle. For *N* = 8, experiments were conducted in eight different wells of cultured cells.

Activation of MMPs is a key factor for BBB breakdown (Kelly et al., 2006; Shigemori et al., 2006). FGF21 itself did not affect the activities of MMP-3 and MMP-9 under normoxia condition (Figures 4A,B, black panel). Upon hypoxia stress, the activities of MMP-3 (Figure 4A) and MMP-9 (Figure 4B) in CMECs displayed 3~5-fold increment. FGF21 treatment significantly decreased the triggered activities of MMP-3 (*p* < 0.01; Figure 4A) and MMP-9 (*p* < 0.01; Figure 4B). These data suggest that FGF21 decreases hypoxia-induced MMP activities in CMECs.

### FGF21 Limits Hypoxia-Induced Inflammation in CMECs

**Figure 5:**
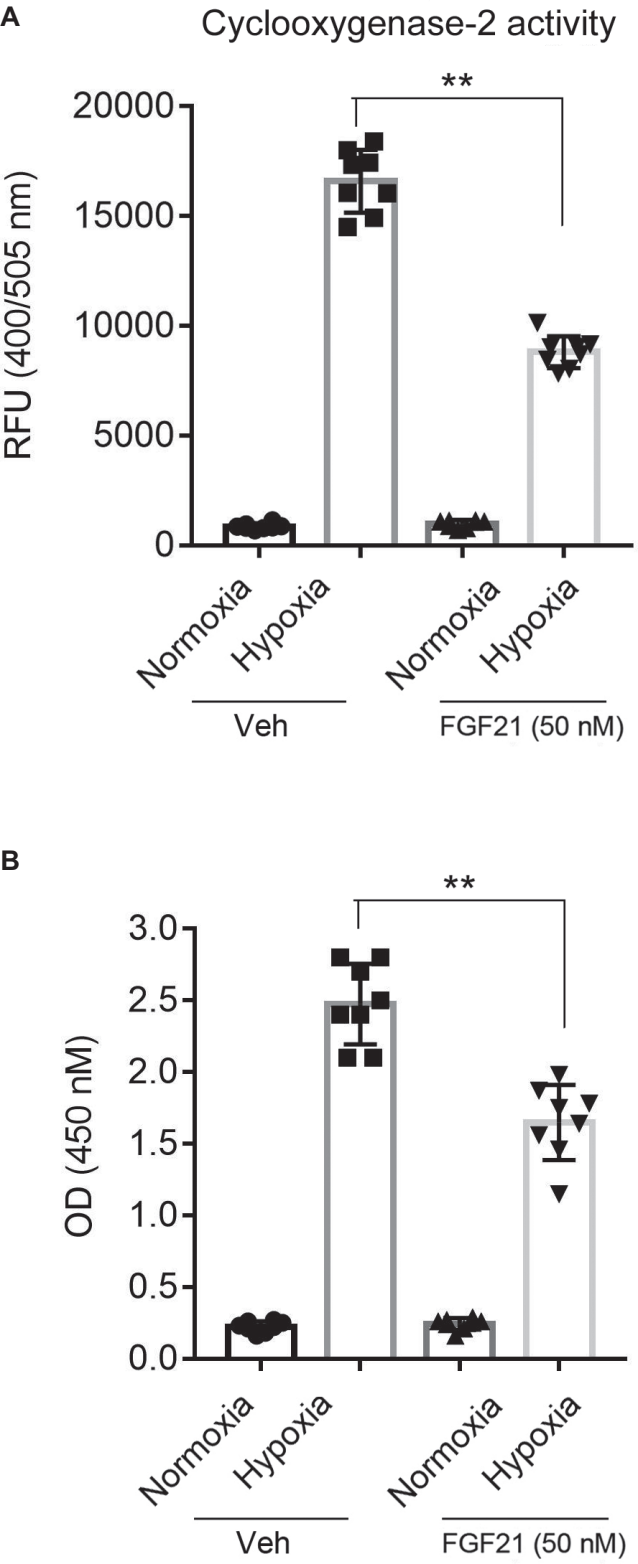
FGF21 limits hypoxia-induced inflammation in CMECs. **(A)** Quantitative analysis of cyclooxygenase-2 (COX-2) activity in CMECs under the condition of normoxia and hypoxia (1% O_2_) with FGF21 treatment (50 nM) or vehicle for 8 h. **p < 0.01 vs. Veh. *N* = 8. **(B)** Quantitative analysis of NF-κB-p65 transcriptional activity in CMECs challenged by hypoxia (1% O_2_) with FGF21 treatment (50 nM) or vehicle for 8 h. **p < 0.01 vs. Veh. *N* = 8. Veh, vehicle. For *N* = 8, experiments were conducted in eight different wells of cultured cells.

COX-2 (Fathali et al., 2010) and NF-κB (Xie et al., 2005) are two pro-inflammatory factors in hypoxia-induced brain injury. So, we next measured the COX-2 activity and the NF-κB-p65 DNA-binding activity in nuclear extracts. The COX-2 activity in hypoxia-treated CMECs increased about ~10 folds compared with that in normoxia (Figure 5A). FGF21 treatment partly reduced the hypoxia-induced COX-2 activity (*p* < 0.01; Figure 5A). Similar results were observed in NF-κB-p65 transcriptional activity assay. Hypoxia stimuli markedly upregulated the NF-κB-p65 transcriptional activity, which was partly suppressed in FGF21-treated CMECs (*p* < 0.01; Figure 5B). These results point out that FGF21 limits hypoxia-induced inflammation in CMECs.

### Blockade of HSP72 by Chemical Inhibitor Compromises the Protection of FGF21 Against Hypoxia in CMECs

**Figure 6:**
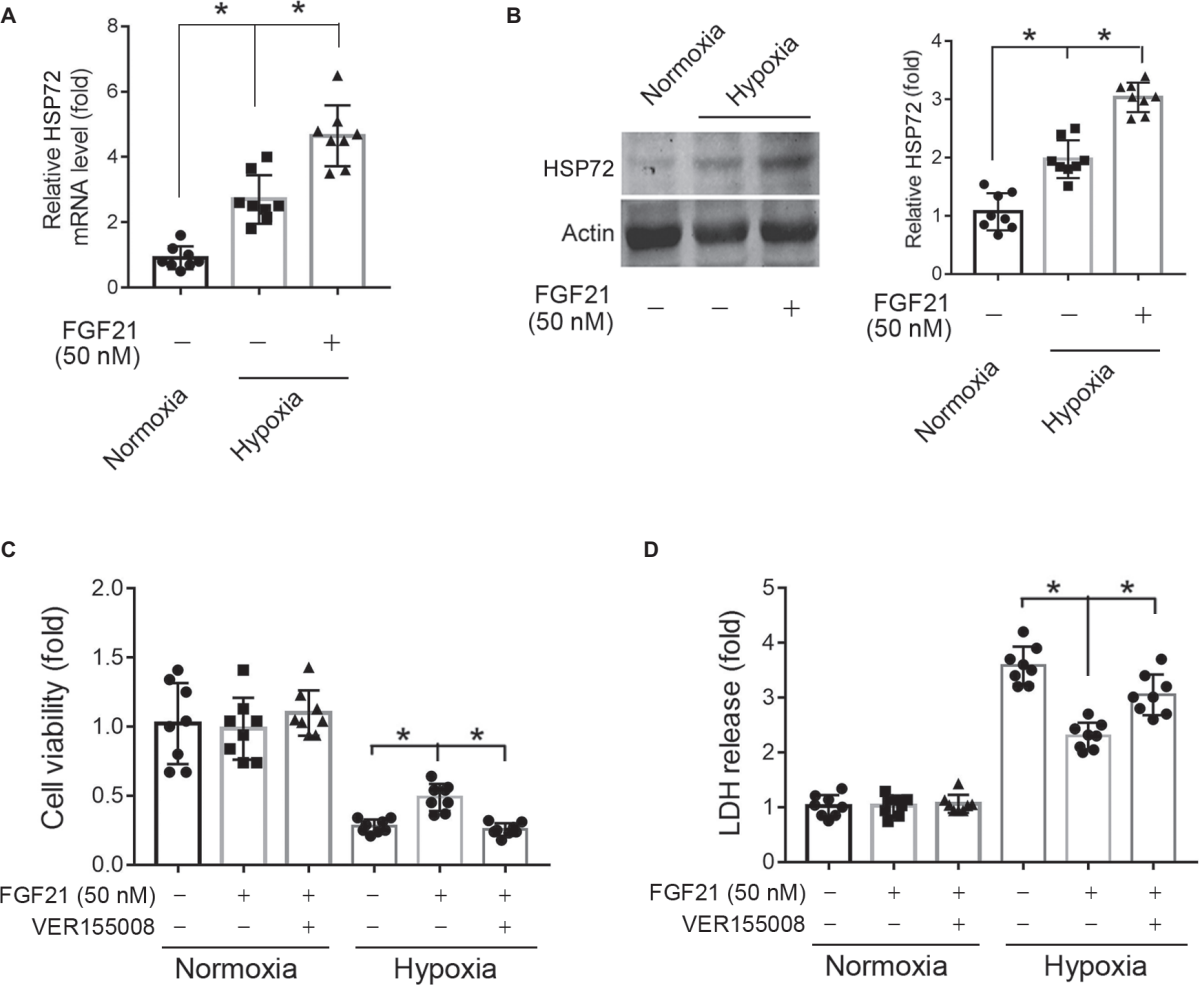
Blockade of HSP72 by chemical inhibitor compromises the protection of FGF21 against hypoxia in CMECs. **(A,B)** The mRNA **(A)** and protein **(B)** levels of HSP72 in CMECs under the condition of normoxia and hypoxia (1% O_2_) with FGF21 treatment (50 nM) or vehicle for 8 h. *p < 0.05. *N* = 8. **(C,D)** The cell viability **(C)** and LDH release **(D)** of CMECs under the condition of normoxia and hypoxia (1% O_2_) with FGF21 treatment (50 nM) or FGF21 + VER155008 (50 μM) or vehicle for 8 h. *p < 0.05. *N* = 8. Veh, vehicle. For *N* = 8, experiments were conducted in eight different wells of cultured cells.

HSP72, a molecular chaperone and is known to prevent incorrect protein folding and apoptosis, has been shown to protect heart and neural system from ischemic injury (Hamilton et al., 2003; Xu et al., 2010). We found that hypoxia treatment for 8 h significantly upregulated HSP72 protein expression in CMECs (*p* < 0.05; Figures 6A,B). This upregulation of HSP72 was further strengthened by FGF21 treatment (*p* < 0.05; Figures 6A,B). To verify the importance of HSP72 modulation in the protection of FGF21, we used VER155008, a selective inhibitor of HSP72, to see whether this compound can block the action of FGF21. We found that VER155008 successfully abolished the protection of FGF21 on cell viability (*p* < 0.05; Figure 6C) and LDH release (*p* < 0.05; Figure 6D) upon hypoxia stress in CMECs. These suggest that HSP72 is important for the protection of FGF21 against hypoxia in CMECs.

### Knockdown of HSP72 Abolishes the Protection of FGF21 Against Hypoxia in CMECs

**Figure 7:**
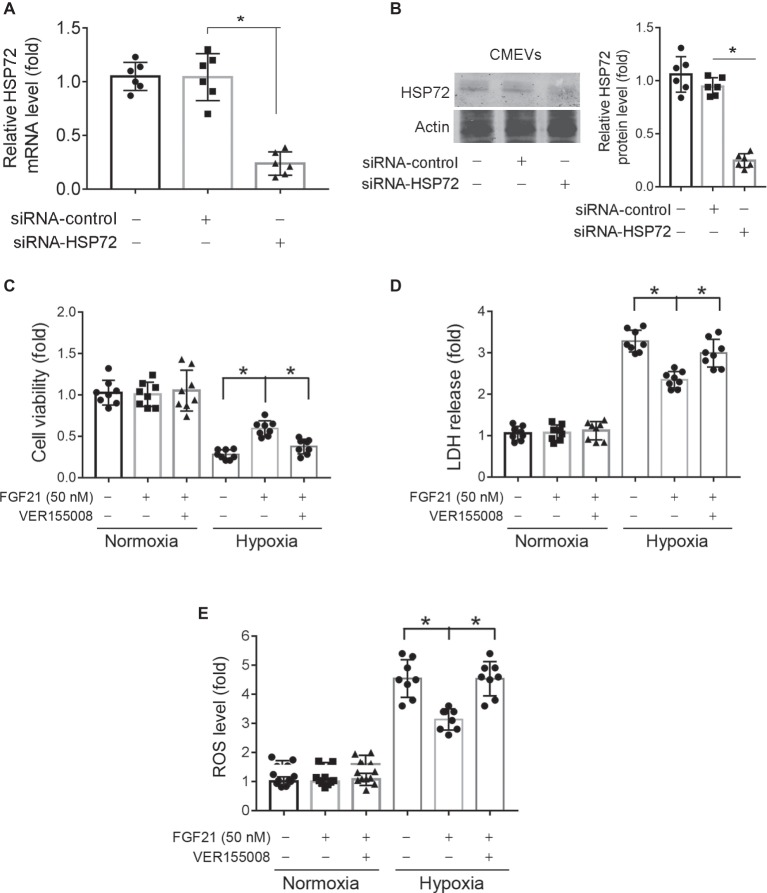
Knockdown of HSP72 abolishes the protection of FGF21 against hypoxia in CMECs. **(A,B)** The effects of siRNA targeting to HSP72 on the mRNA **(A)** and protein **(B)** expression of HSP72 in CMECs. *p < 0.05. *N* = 6. Actin was used as a loading control. **(C,E)** The cell viability **(C)**, LDH release **(D)**, and ROS **(E)** of normal or HSP72-knockdown CMECs under the condition of normoxia and hypoxia (1% O_2_) with FGF21 treatment (50 nM) for 8 h. *p < 0.05. *N* = 8. Veh, vehicle. For *N* = 6 or 8, experiments were conducted in eight different wells of cultured cells.

We also applied siRNA-mediated knockdown to verify the importance of HSP72 in protection of FGF21 against hypoxia in CMECs. Specific siRNA targeting HSP72 successfully reduced HSP72 mRNA (Figure 7A) and protein (Figure 7B) expression in CMECs. Knockdown of HSP72 did not display any effects on cell viability and LDH release under normoxia condition (Figures 7C,D). However, knockdown of HSP72 partly abolished the protection of FGF21 on cell viability (*p* < 0.05; Figure 7D) and LDH release (*p* < 0.05; Figure 7E) in CMECs upon hypoxia stimuli. At last, knockdown of HSP72 prevented the action of FGF21 on ROS production under hypoxia stimuli (*p* < 0.05; Figure 7E). All these results further support that HSP72 is a mediator of the protection of FGF21 against hypoxia in CMECs.

## Discussion

In the present study, we provided the first evidence that administration of FGF21 attenuates hypoxia-induced injury in CMECs. We showed that FGF21 treatment not only inhibited the hypoxia-induced cell apoptosis/death but also substantially prevented hypoxia-induced oxidative stress in cultured CMECs. Additionally, FGF21 exhibited potent suppression on activities of MMP-3 and MMP-2/9. In support of this, the hypoxia-induced COX-2 activity and NF-κB-p65 transcriptional capacity were also inhibited by FGF21. At last, we found FGF21 upregulated HSP72 mRNA and protein expression, while blockade of HSP72 by pharmacological inhibitor VER155008 or siRNA-mediated knockdown abrogated the protection of FGF21 against hypoxia in CMECs.

The first important finding in our study is that we found that FGF21 is a protector against hypoxia stress in CMECs. Recombinant FGF21 has been used for metabolic disease therapy in human. Treatment of engineered recombinant FGF21 (LY2405319) for 1 month has been tested for its improvement of LDL-C, HDL-C, triglycerides, body weight, blood adiponectin, and β-hydroxybutyrate levels in obese patients with type 2 diabetes (Gaich et al., 2013). Some previous investigations have pointed out that FGF21 displayed neuroprotection against ischemic injury. Yang et al. reported that administration of FGF21 reduced infarcted area and decreased ER stress in cerebral ischemic rats by its neuroprotection (Yang et al., 2018). It should be noted that hypoxia stress is not only one of the most well-known causes of ischemic damage but also crucial pathophysiological factors of other hypoxia-associated diseases, including high-altitude cerebral edema, acute mountain sickness, and diving-related brain injury (Bailey et al., 2009; Gren et al., 2016). All these diseases involve hypoxia-induced CMEC injury and BBB disruption. Our results are in line with those in two very recent published papers. Yu et al. reported that FGF21 treatment significantly ameliorated diabetes-induced BBB permeability and preserved junction protein expression *in vivo* (Yu et al., 2018). Another group showed that FGF21 protected BBB by upregulating PPARγ *via* FGFR1/β-Klotho in traumatic brain injury (Chen et al., 2018). The BBB disruption caused by diabetes and traumatic brain injury may somehow differ from hypoxia-induced BBB disruption. The receptor of FGF21 is a complex, which is composed of FGF receptor 1c (FGFR1c) and β-Klotho. These factors are expressed in endothelial cells (Asashima et al., 2003). Moreover, they are upregulated and also display protection against hypoxia/ischemia injury (Muinck et al., 2007; Zhou et al., 2017a). Nevertheless, our data and these researches strongly imply the therapeutic value of FGF21 in brain diseases with severe BBB disruption. Giving that engineered FGF21 analog has been successfully applied for diabetes treatment in clinical (Gaich et al., 2013), our results may extend the indication of the engineered FGF21 analog from metabolic disorders to hypoxia-related brain diseases such as brain ischemia and acute mountain sickness. A recent study showed that administration of lyophilized FGF21 protected cerebral ischemia in rats subjected with brain ischemia and neuron cell line (Yang et al., 2018). Moreover, peripherally derived FGF21 promotes remyelination in the central nervous system (Kuroda et al., 2017). Consequently, it is very likely that FGF21 also has protective effects in non-cerebral endothelial cells under ischemic stress.

The second important finding of our study is that FGF21 induces HSP72 expression upon hypoxia. HSP72 is one of the most important members of HSP70 family proteins. As a major inducible HSP, HSP72 plays important roles in many fundamental cellular stress-related activities including protein synthesis, folding, translocation, interaction, and degradation (Clerico et al., 2015). HSP72 also contributes to the complex pathophysiological mechanisms of diseases such as cancer (Gabai et al., 2009), inflammatory bowel diseases (Samborski and Grzymislawski, 2015), and insulin resistance (Drew et al., 2014). Specifically, HSP72 is rapidly expressed in response to hypoxia stimuli (Taylor et al., 2010), and hydrogen peroxide could induce HSP72 and thus resulted in apoptosis in hypoxic endothelial cells (Motoyama et al., 2000). HSP72, but not HSP90α, is the major effector underlying the beneficial action of heat acclimation in acute hypoxia (Gibson et al., 2015). Moreover, human monocyte HSP72 was greatly induced in responses to acute hypoxic exercise (Lee et al., 2015). These results highlight the potential roles of HSP72 in hypoxia-related diseases. Indeed, several works have uncovered the protective effects of HSP72 in hypoxia-related diseases. Overexpression of HSP72 preserved renal function in kidney ischemic/reperfusion injury (Zhang et al., 2015). HSP72 also improved long-term recovery (Xu et al., 2011) and inhibited JNK-dependent neuronal apoptosis during cerebral ischemia (Qi et al., 2012). As FGF21 upregulated HSP72 mRNA and protein, we considered that the regulation of FGF21 on HSP72 is on transcriptional level. Another factor should be taken into account is peroxisome proliferator-activated receptor gamma (PPAR-γ). Upregulation of PPAR-γ is responsible for many effects of FGF21 (Hui et al., 2016; Liu et al., 2018). Interestingly, there may be a close relationship between PPAR-γ and HSP72 because naringin upregulates PPARγ and HSP72 simultaneously (Sharma et al., 2011). Considering that HSP72 and PPARs form a complex *in vivo* and that HSP72 participates in the folding, subcellular localization, and/or signaling pathway of PPARs (Huang et al., 1994), we think PPARγ may play a role in the regulation of FGF21 on HSP72. Nevertheless, this speculation needs more investigation in the future.

There may be some limitations in our study. First, we only tested one dose of FGF21 in CMECs. The dose of FGF21 used in our work is 50 nM, which is very similar to the physiological levels of FGF21 in blood. The FGF21 level in serum was reported to be 16~50 nM (Galman et al., 2008). However, the FGF21 levels in blood may increase by several folds to reach ~250 nM in some diseases such as chronic hemodialysis (Stein et al., 2009). Thus, the circulating FGF21 may directly exert its biological function on BBB. The second limitation is that we used a cell line but not animal model to test the protection of FGF21 on BBB. Since other cells, such as astrocytes and microglia, also contribute to the BBB maintenance, further work on the cell-cell interaction during BBB disruption should be considered. At last, our work did not explore the interactions between the markers such as ROS, MMPs, COX, and NF-κB. We considered that hypoxia might induce massive intracellular responses in CMECs. Shin et al. demonstrated that ROS can induce MMP-1 and -9 expressions *via* activating MAPKs (Shin et al., 2008). There are also some works showing that ROS is an upstream event of COX-2 and NF-κB induction (Yang et al., 2011; He et al., 2014). Thus, the inhibition of FGF21 on ROS production may be the primary event for the protection of FGF21 against MMPs, COX-2, and NF-κB.

In conclusion, we demonstrate that FGF21 protects against hypoxia stress-induced injury in CMECs, evidenced by the improvement of FGF21 on apoptosis, oxidative stress, MMP activity, COX-2 activity, and NF-κB-p65 transcription factor activity. Moreover, we provide evidence that induction of HSP72 by FGF21 contributes to the protection of FGF21. Our results indicate that FGF21 may be a promising therapeutic candidate for patients with hypoxia-related BBB disruption and brain diseases.

## Author Contributions

H-WW, XJ, YZ, JW, and JX performed experiments and analyzed the data. Y-QW and Y-HL received funding and drafted and revised the manuscript.

### Conflict of Interest Statement

The authors declare that the research was conducted in the absence of any commercial or financial relationships that could be construed as a potential conflict of interest.
